# Soft Selective Sweeps in Evolutionary Rescue

**DOI:** 10.1534/genetics.116.191478

**Published:** 2017-02-17

**Authors:** Benjamin A. Wilson, Pleuni S. Pennings, Dmitri A. Petrov

**Affiliations:** * Department of Biology, Stanford University, California 94305; † Department of Biology, San Francisco State University, California 94132

**Keywords:** adaptation, extinction, population density, genetic diversity, resistance evolution

## Abstract

Evolutionary rescue occurs when a population that is declining in size because of an environmental change is rescued from extinction by genetic adaptation. Evolutionary rescue is an important phenomenon at the intersection of ecology and population genetics, and the study of evolutionary rescue is critical to understanding processes ranging from species conservation to the evolution of drug and pesticide resistance. While most population-genetic models of evolutionary rescue focus on estimating the probability of rescue, we focus on whether one or more adaptive lineages contribute to evolutionary rescue. We find that when evolutionary rescue is likely, it is often driven by soft selective sweeps where multiple adaptive mutations spread through the population simultaneously. We give full analytic results for the probability of evolutionary rescue and the probability that evolutionary rescue occurs via soft selective sweeps. We expect that these results will find utility in understanding the genetic signatures associated with various evolutionary rescue scenarios in large populations, such as the evolution of drug resistance in viral, bacterial, or eukaryotic pathogens.

ABRUPT environmental changes may lead to a demographic decline in a population when the population is maladapted to the new environment. This scenario necessitates an evolutionary response from the population if the population is to escape extinction. The process by which genetic adaptation allows a population to recover from the demographic consequences of harsh environmental shifts has been termed “evolutionary rescue.” Evolutionary rescue is a focus of many studies at the interface of ecology and evolution in part due to recent attention to climate change, drug resistance, pesticide resistance, and other anthropogenic change of global and local environments (Gonzalez *et al.* 2013; Alexander *et al.* 2014; Carlson *et al.* 2014). While much attention has been given to theoretical and experimental predictions regarding the probability that evolutionary rescue occurs under various scenarios, we focus on the dynamics of adaptive alleles and the associated genetic signatures left behind by adaptation. More explicitly, we are interested in how selective sweeps that enable evolutionary rescue differ in the number of ancestral backgrounds on which they emerge, based on the conditions under which evolutionary rescue occurs. If evolutionary rescue is facilitated by “hard” selective sweeps—wherein a single progenitor is responsible for the spread of the beneficial variant—then genetic diversity will be removed from the population as a result of adaptation as well as demographic decline. By contrast, a population that adapts via “soft” selective sweeps—wherein multiple ancestors have independently derived beneficial variants—will preserve some of the ancestral diversity that was present prior to the environmental shift that caused the population to decline (Pennings and Hermisson 2006b).

Soft selective sweeps occur when adaptive alleles appear in multiple individuals prior to sweeping through the population. This leads to a sample genealogy that includes multiple adapted ancestors in the new environment. The underlying criterion for the occurrence of soft selective sweeps is that the presence of adaptive mutations in the population is not a limiting factor to the process of adaptation (Hermisson and Pennings 2005; Pennings and Hermisson 2006a; Burke 2012; Messer and Petrov 2013). This criterion is fulfilled in many situations, such as adaptation from previously neutral or deleterious standing genetic variation (Hermisson and Pennings 2005) and adaptation from recurrent *de novo* mutations (Pennings and Hermisson 2006a) in large populations. While soft selective sweeps appear to be abundant in case studies of adaptation (Messer and Petrov 2013), the signature of a soft selective sweep is crucially dependent on the population sample and the underlying demographic history of the population from which the sample is taken. Importantly, many case studies of adaptation pertain to situations where the demography of the population and the process of adaptation are necessarily interrelated. Particularly in the case of resistance evolution, beneficial mutations of large effect often confer benefits in terms of absolute fitness rather than relative fitness; thereby leading to demographic changes to the population. Previous work suggests that soft selective sweeps in populations with fluctuating population size can give rise to signatures of both hard and soft sweeps depending on when beneficial alleles arise, when they are sampled, and how advantageous they are (Wilson *et al.* 2014). However, in that work we had assumed that demography was independent of the allelic state at the locus under selection, an assumption that is valid only under models where fitness advantages are relative and density independent (*e.g.*, the standard Wright–Fisher model with selection or the Moran model). In this article, we allow adaptation to influence demography. We explore a simple logistic model where fitness advantages are absolute and density dependent within the context of an evolutionary rescue scenario. While most models of evolutionary rescue predict the probability that rescue occurs (Gomulkiewicz and Holt 1995; Orr and Unckless 2008, 2014; Uecker and Hermisson 2011, 2016; Martin *et al.* 2013; Uecker *et al.* 2014), we focus on the likelihood that rescue occurs via hard or soft sweeps.

The primary result of our analysis is that when rescue is likely to occur, it is more likely to occur via soft selective sweeps than hard selective sweeps. This result follows intuitively from the observation that the higher the time-averaged input of adaptive mutations, the greater the probability of evolutionary rescue as well as the probability of soft selective sweeps. We demonstrate how our result is critically dependent on the population-scale mutation rate at the onset of the wild-type population decline, and whether mutant growth rates are restricted by population density. We give analytical results for the probability of evolutionary rescue and the probability that rescue occurs via soft selective sweeps. We also give the waiting-time distributions for rescuing beneficial mutations. In the context of previous results connecting soft sweeps and postadaptation genetic diversity, our results highlight a key correlation between genetic diversity following evolutionary rescue and the likelihood of evolutionary rescue (Feder *et al.* 2016).

## Methods

### Simulations

Simulations were performed using a Gillespie algorithm (Gillespie 1976) programmed in Python (Python Software Foundation, Python Language Reference, version 2.7.6, available at http://www.python.org). All simulations began at the onset of the wild-type population decline. The wild-type population was initialized at a population size of $10^{4}$ to be large enough to model the evolutionary processes by continuous approximations, but small enough to be computationally efficient. For each round of simulation in the birth-death process, the algorithm used went as follows:

1. Sample the waiting time *t* for an event from an exponential distribution with rate parameter equal to the sum of all rates for all possible events beginning at time 0.
2. Randomly assign a specific event according to the relative probabilities of occurrence of each type of event (*i.e.*, mutation, wild-type birth/death, mutant birth/death).
3. Then update the population and all event rates for the new time *t*.

The process was repeated until either (1) the population went extinct and no rescue occurred, or (2) adaptation successfully rescued the population and the new mutant population reached 99% of its equilibrium size. Note that in simulations where rescue occurred, we did not necessarily wait until the mutant subpopulation reached fixation. This was done to model the effects of sampling the population when rescue would likely be suspected, rather than when complete replacement of the wild-type population occurred; a feature that we believe to be more realistic. At the end of each simulation, the population composition was analyzed to determine if (1) no lineages existed following an extinction event; (2) only one mutant lineage existed, indicating a hard selective sweep; or (3) more than one lineage existed, indicating a soft selective sweep.

To encapsulate the extremes of the parameter range, we simulated all combinations of the following:

1. Five different wild-type decline rates: α∈{0.01,0.03,0.1,0.3,1.0}.
2. Three different mutation rates: $\mu \in \left\{10^{-5},10^{-4},10^{-3}\right\}.$
3. Three different mutant birth rates: $b_{m}\in \left\{1.1,1.3,2.0\right\}.$
4. Two different population-size limits: K∈{10,000, 110,000}.

For this particular range of *α* from 0.01 to 1.0, wild-type populations went extinct by approximately $\tau_{\text{end}}=\log\left(w_{0}\right)/\alpha$ which ranged from slow declines of length $\tau_{\text{end}}=1000$ to extremely rapid declines of length $\tau_{\text{end}}=10,$ respectively. We restricted birth rates to be relatively small to prevent biologically unrealistic dynamics in the mutant population growth; however, the absolute growth rates are potentially large at low population density. For all simulations, the wild-type and mutant death rates were set to one. The population-size limits were chosen to produce scenarios where the mutant would be unconditionally beneficial from the onset of the population decline (K=110,000) and where the mutant would initially suffer growth costs at high population density (K=10,000). Note that in the latter case, the mutant is actually deleterious with respect to the wild type for all *α* except α=1.0, in which case the mutant and wild type are initially identical in fitness. The parameter ranges were chosen to cover a wide range of phenomena, with combinations that produced extinction almost always and combinations that produced rescue via soft selective sweeps almost always.

### Analysis

All mathematical analysis was numerically evaluated in Mathematica (Wolfram Research, Inc. 2010). In practice, we found it neither necessary nor biologically meaningful to integrate to long times for all of our numerical integrations, so we chose to integrate to the time when a deterministically declining wild-type population would reach a single individual, $\tau_{\text{end}}=\log\left(w_{0}\right)/\alpha ,$ for our analytic calculations of $P_{\text{rescue}}$ and $P_{\text{soft}}.$ For the waiting-time distributions for $\tau_{1}$ and $\tau_{2},$ we chose to integrate to the time when a deterministically declining wild-type population would reach 10% of its original size, $\tau_{\text{end}}=\log\left(w_{0}/1000\right)/\alpha .$

### Data availability

All code for the work performed in this article is available online at: https://github.com/benwilson87/evolutionary-rescue-soft-sweeps. All plots were made using the ggplot2 (Wickham 2009) package in R (R Core Team 2015).

## Results

We begin by modeling a population that is going extinct because of an environmental shift that leads to a demographic decline in the population (*e.g.*, a drug enters the host environment of a virus causing the viral population to crash). We assume that mutants are not present at the onset of the population decline, *i.e.*, there are no adaptive mutations present as standing genetic variation (*e.g.*, because the adaptive mutations conferring drug resistance are highly deleterious in the absence of the drug). Adaptive mutations emerge on the background of the maladapted wild type. We assume a single-locus, two-allele model where the mutation rate toward the beneficial state is *μ* and is constant in time. We assume back mutations to the wild-type state are negligible. Individuals of the two types, maladapted wild type (*w*) and adapted mutant (*m*), give birth or die with transition rates given by

$w\to w+1:b_{w}w,$
$w\to w-1:d_{w}w,$
$m\to m+1:b_{m}m\left[1-\left(m+w\right)/K\right],$ and
$m\to m-1:d_{m}m;$

where we assume *m* + *w* < *K* so that mutant birth rates are strictly positive and biologically meaningful. The decline of the wild-type (maladapted) population is intrinsic to the genotype and is density independent, *i.e.*, the wild type suffers decreased reproductive success directly from its interaction with the environment and not from competition for shared resources. The wild-type population size can be deterministically approximated by $w\left(t\right)=w_{0}\exp\left(-\alpha t\right),$ where the variable *α* sets the rate at which the wild-type population declines and is equivalent to the absolute difference in per-capita birth and death rates $(\alpha =d_{w}-b_{w}>0,$ to maintain the sign convention previously presented). It is worth noting that Orr and Unckless (2008) found the probability of evolutionary rescue to be approximately twofold higher for wild-type populations that experience logistic population regulation rather than the strictly exponential decline in a previous model; however, we will focus strictly on the case of exponential decline for model simplicity. The carrying capacity *K* sets the scale of density dependence and determines the equilibrium population size for the adapted population should adaptation occur: $m_{\text{eq}.}=K\left(1-d_{m}/b_{m}\right),$ which is the value of *m* obtained by setting the mutant birth rate $b_{m}m\left[1-\left(m+w\right)/K\right]$ equal to the mutant death rate $d_{m}m$, setting w=0, and solving for *m* assuming $b_{m}>d_{m}.$

For simplicity, we will assume that $d_{m}=d_{w}$ such that the expected lifetime of each type is the same. In an alternative model, this assumption could be relaxed to investigate how genetic alterations to generation time or how different reproductive strategies (such as viral latency) could facilitate evolutionary rescue, but we will not explore these scenarios in this investigation. Ignoring the density-dependent scaling factor, 1−(m+w)/K, the difference $b_{m}-b_{w}$ could be interpreted as the genotype-intrinsic growth advantage of a mutant individual over a wild-type individual. The parameter $b_{m}$ also sets the maximum per-capita birth rate for mutants at low population density. For our model, we do not consider extremely large birth rates $\left(b_{m}\gg 1\right)$ to avoid extreme jumps in the population size over short timescales. Note that this model is closely analogous to scenario 2 under “Alternative Forms of Population Regulation” in Orr and Unckless (2008), except that our model is in continuous time rather than discrete time. Our model is also similar to the D=1 panmictic model presented in Uecker *et al.* (2014), except that we exclude the contribution of standing genetic variation and again look at continuous time *vs.* discrete time.

**Figure 1:**
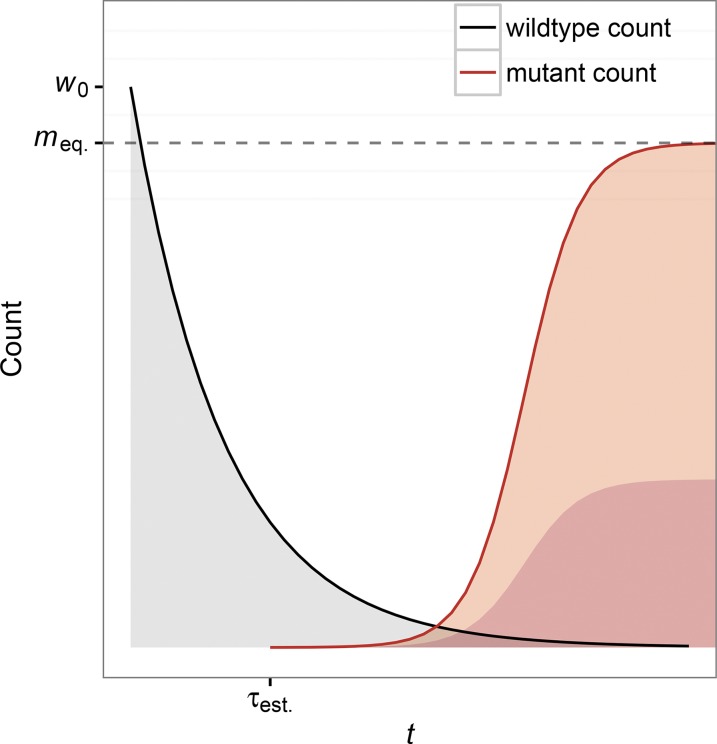
Model depiction of evolutionary rescue. A population initially comprised of maladapted wild-type individuals (black line) declines exponentially from its original size $w_{0}$ following an environmental shift. A beneficial mutation appears on the background of a wild-type individual and establishes at time $\tau_{\text{est}.},$ at which point the population is destined to be rescued via adaptation. If a mutant fails to establish before the wild-type population goes extinct, then no rescue occurs. Following rescue, the mutant population (red line) will equilibrate at a new population size $m_{\text{eq}.}=K\left(1-d_{m}/b_{m}\right).$ In some cases of rescue, multiple mutant lineages can establish before the wild-type population goes extinct, leading to a soft selective sweep, as illustrated by the multiple shaded lineages (red shading) within the mutant population (red line).

At any given time *t*, mutations occur at a rate w(t)μ and establish with a probability $p_{\text{est}.}\left(t\right).$ Establishment occurs when a mutation survives extinction due to drift at low copy number. From analysis of soft sweeps via *de novo* mutation in populations of constant size, we know that soft sweeps are only expected to occur when $w_{0}\mu ∼1$ or greater (Pennings and Hermisson 2006a). In most scenarios we consider, we correspondingly scale *μ* to be either $1/w_{0}$ or $10/w_{0}$ to ensure that mutations occur frequently enough at the beginning of the environmental shift to expect multiple adaptive lineages to appear during the rescue scenario; though their survival will ultimately depend on the other parameters previously described, namely the carrying capacity (*K*), the wild-type decline rate (*α*), and the mutant birth rate $\left(b_{m}\right).$ Note that the decline in the wild-type population means that adaptation will eventually be mutation limited in all cases, *i.e.*, the supply rate of mutations will always go to zero as the wild-type population goes extinct. We also consider situations where adaptation is likely to only proceed via hard sweeps (if at all) $\left(w_{0}\mu <1\right)$ to illustrate the limitations of our results. Figure 1 gives an illustration of the rescue scenario.

### The time-dependent probability of establishment

We can derive the probability of establishment for a mutation arising at a particular time *τ* using the methodology presented in Uecker and Hermisson (2011) (see specifically equation A5 under “Fixation in General Ecological Models” in the appendix and equation 16a in the main text, as well as Allen 2010, pp. 278–280, for the general theory). Uecker and Hermisson (2011) showed that for a time-inhomogeneous birth-death process (such as the specific birth-death model presented here) we can write the probability of establishment for a single mutant starting at particular time τ=0 in terms of the total per-capita mutant birth rate B(t) and total per-capita mutant death rate D(t). The general result takes the form

$$p_{\mathrm{est}.}=\frac{2}{1+\int_{0}^{\infty }\left[B\left(t\right)+D\left(t\right)\right]\exp\left\{-\int_{0}^{t}\left[B\left(t^{\prime }\right)-D\left(t^{\prime }\right)\right]dt^{\prime }\right\}dt},$$

where $t^{\prime }$ is a dummy variable for the nested integral. For our specific birth-death model, $B\left(t\right)=b_{m}\left\{1-\left[w\left(t\right)\right]/K\right\}$ and $D\left(t\right)=d_{m}$ can be taken directly from the transition rates presented at the beginning of this section, and the probability of establishment for a single mutant appearing at a particular time *τ* is given by

$$p_{\mathrm{est}.}\left(\tau \right)=\frac{2}{1+\int_{0}^{\infty }\left\{b_{m}\left[1-\frac{w\left(t+\tau \right)}{K}\right]+d_{m}\right\}\exp\left(-\int_{0}^{t}\left\{b_{m}\left[1-\frac{w\left(t^{\prime }+\tau \right)}{K}\right]-d_{m}\right\}dt^{\prime }\right)dt},$$ (1)

where the instantaneous time *τ* now appears explicitly because *τ* is not fixed at zero.

Note that we have neglected the mutant population size in the density-dependent terms under the assumption that mutant lineages have independent probabilities of establishment while the mutant population size is low and while the expected time between successive establishments is short. Later we will show that this assumption breaks down for rescue scenarios with slow decline rates and when the expected time between mutant establishment increases.

### The role of population density

We demonstrate here how population density influences the process of evolutionary rescue in our model. Note that population density is the population size relative to the carrying capacity, not the population size itself. Including density dependence through a carrying capacity *K* ensures that a rescued population reaches an equilibrium size at long timescales. Population density also has critical effects on mutant establishment because it determines the growth rate of the mutant through time.

We can separate the effects of population density into two characteristics: the growth rate of the mutant at the onset of the wild-type population decline and the rate at which density-dependent growth restriction decays over time. The initial growth rate of the mutant depends on the ratio of the starting wild-type population size to the total population-size limit $\left(w_{0}/K\right).$ If the initial population density is high $\left(w_{0}/K>1-d_{m}/b_{m}\right),$ *i.e.*, the wild-type population size is similar to the carrying capacity, then the birth rate of the mutant may (initially) be lower than its death rate, making it unlikely that a mutant establishes.

By contrast, in situations where the carrying capacity is much larger than the wild-type population size $\left(w_{0}/K<1-d_{m}/b_{m}\right),$ a mutant lineage that appears at the onset of the wild-type population decline will have a net positive growth rate from the onset. In other words, early mutants have less chance of surviving when initial density is higher.

The rate of decline of the wild-type population determines how quickly mutant growth rates increase. Scenarios with fast wild-type population decline will alleviate mutant growth restrictions more quickly and increase the probability of establishment (conditional on appearance), although fast wild-type population decline will also decrease the rate at which mutants appear. We highlight these different aspects of density dependence in our model because we find it important to note that (1) early mutants are not unconditionally advantageous compared to wild-type individuals, and (2) the probability of establishment is intrinsically tied to the wild-type population decline. While this first point arises mathematically from our assumption of wild-type population decline being density independent, we retain it as a mathematical convenience to model a mutant fitness trade-off between rapid growth at low population density and weak competitive ability at high population density without the addition of another model parameter.

**Figure 2:**
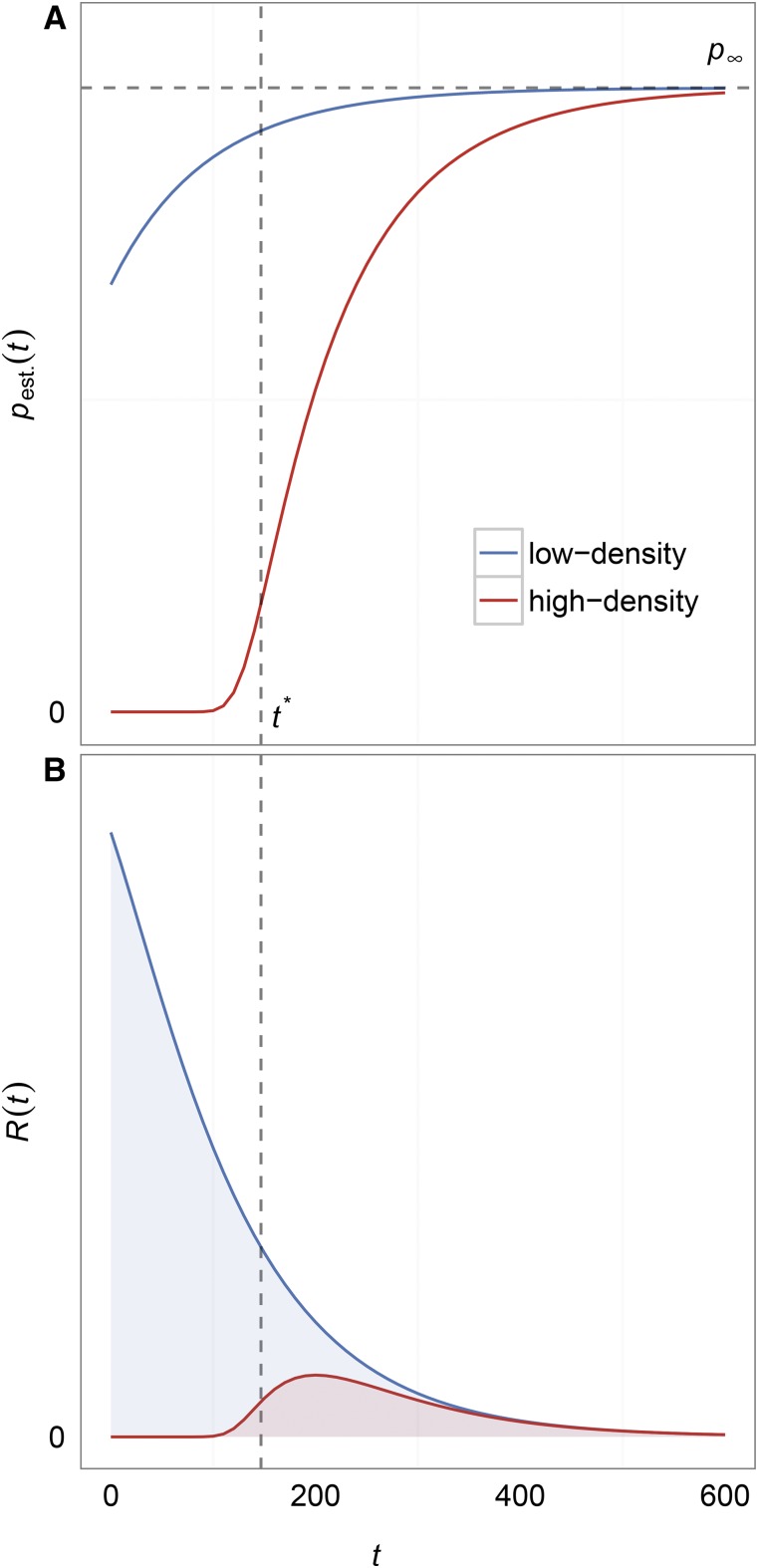
Establishment probability and intensity function distributions. (A) The establishment probability distributions for mutations appearing at time *t* in low- (top, blue) and high-density (bottom, red) scenarios with all parameters being equal except for *K*. For low-density scenarios, the probability of establishment increases monotonically as the density restriction on mutant growth declines to zero with the wild-type population. For high-density scenarios, the probability of establishment is essentially zero until the wild-type population declines to a size w* at t* (see Equation 2), whereupon it will increase monotonically just as in the low-density scenario (these approximations can be conservative). Note that both low- and high-density scenarios have distributions that asymptote to the same value, $p_{\infty },$ because $p_{\infty }$ is only dependent on the unscaled per-capita birth and death rates of the mutant (see Equation 3). (B) The distributions for the corresponding intensity functions $R\left(t\right)=w\left(t\right)\mu p_{\text{est}.}\left(t\right)$ for the same two scenarios in (A). R(t) gives the instantaneous rate at which mutants successfully establish and save the population from extinction. R(t) eventually declines to zero with the wild-type population size (see Figure 1) even though the establishment probability increases with time. The shaded area under R(t) determines the probability of evolutionary rescue and is generally larger in low-density (top, blue) scenarios than in high-density scenarios (bottom, red).

The influence of population density on $p_{\text{est}.}\left(t\right)$ can be seen by comparing values of $p_{\text{est}.}\left(t\right)$ between situations of high and low population density at the onset of the wild-type population decline, as illustrated in Figure 2A. In the scenario with high population density $\left(w_{0}/K>1-d_{m}/b_{m}\right),$ $p_{\text{est}.}\left(t\right)$ is essentially zero until the wild-type population has declined sufficiently to give mutants a positive growth rate (bottom, red line). This growth rate transition occurs at $w\left(t\right)=w*=K\left(1-d_{m}/b_{m}\right).$ Using the deterministic approximation to the wild-type population size, we can estimate that this transition occurs at

$$t*∼-\frac{1}{\alpha }\log\left[\frac{K\left(1-d_{m}/b_{m}\right)}{w_{0}}\right]$$ (2)

by setting $w\left(t\right)=w*=K\left(1-d_{m}/b_{m}\right),$ and solving for *t*. By contrast, the scenario with low population density at the onset $\left(w_{0}/K<1-d_{m}/b_{m}\right)$ shows that beneficial mutants have an appreciable probability of establishing from the beginning of the environmental shift (top, blue line). Note that because the probability of establishment is measured for a mutation occurring at time *τ* and because the density restriction imposed by the wild-type population declines monotonically with time, $p_{\text{est}.}$ will increase monotonically with time in our model. It asymptotes at a value

$$p_{\infty }=\frac{b_{m}-d_{m}}{b_{m}}$$ (3)

which is obtained by taking the limit as τ→∞ for Equation 1 and is therefore independent of *K* and *α* because the wild-type population will eventually go extinct. When we set $d_{m}=1$ and when the mutant birth rate is not much higher than 1, we find that the fixation probability is $∼b_{m}-1,$ which is equivalent to *s* in a constant population size model or s+r in a growing population. The reason we do not recover the classic $p_{\text{est}.}\approx 2s$ result from Haldane (1927) or the 2(s+r) result from Otto and Whitlock (1997) is that in our model (with $d_{m}=1,$ $b_{m}=1+s,$ and *s* small) the variance in offspring number is twice what it is in a Wright–Fisher model, which means that the fixation probability is half of what it is in a Wright–Fisher model.

Although the probability of establishment increases over time, the rate of appearance of mutants [w(t)μ] decreases over time, coinciding with the wild-type population size decline. Thus, the total rate of successful beneficial mutants, $R\left(t\right)=w\left(t\right)\mu p_{\text{est}.}\left(t\right),$ will eventually decay as shown in Figure 2B. In the following section, we show how R(t) can be used as the intensity function for a time-inhomogeneous Poisson process that determines mutant establishments.

### Evolutionary rescue via soft selective sweeps

With these model considerations in mind, we can derive the probability that a population headed for extinction is rescued by at least one successful adaptive mutant. If we model mutant establishments using a time-inhomogeneous Poisson process with rate parameter Λ, the general form of the probability that *k* mutants establish is $P\left(K=k\right)=\Lambda^{k}\exp\left(-\Lambda \right)/k!,$ assuming independence. For our model, the rate parameter is derived by integrating the intensity function $R\left(t\right)=w\left(t\right)\mu p_{\text{est}.}\left(t\right)$ over all time. The probability of rescue is $1-P_{\text{extinction}},$ or one minus the probability that no mutants establish (k=0). The probability that no mutants establish is $\exp\left[-\int_{0}^{\infty }R\left(t\right)dt\right].$ This leads to a total probability of rescue equal to

$$P_{\text{rescue}}=1-\exp\left[-\int_{0}^{\tau_{\text{end}}}R\left(t\right)dt\right],$$ (4)

where we have replaced the upper limit ∞ with $\tau_{\text{end}}=\log\left(w_{0}\right)/\alpha ,$ representing the time it would take for a deterministically declining wild-type population to reach a single individual. Note that this is the same result as obtained by Uecker and Hermisson (2011) in equation A7. The integral in Equation 4 is the area under the intensity function depicted in Figure 2B and represents the number of mutants expected to establish during the time when mutations can occur in wild-type individuals.

Assuming independence between mutant lineages, we can gain an overall picture of whether rescue is more likely to occur via hard or soft selective sweeps using the same time-inhomogeneous Poisson process to model the establishment of each individual lineage. To determine the probability of evolutionary rescue via soft selective sweeps, we will first want to calculate that the probability that only one mutant establishes (k=1) before the wild-type population goes extinct, *i.e.*, evolutionary rescue occurs via a hard selective sweep. This is given by

$$P_{\text{hard}}=\left[\int_{0}^{\tau_{\text{end}}}R\left(t\right)dt\right]\exp\left[-\int_{0}^{\tau_{\text{end}}}R\left(t\right)dt\right].$$ (5)

Evolutionary rescue requires at least one mutant lineage to establish before the wild-type population goes extinct, and all evolutionary rescue that does not occur via a hard sweep must occur via a soft sweep by definition. Therefore, the probability of evolutionary rescue via soft selective sweeps is

$$P_{\text{soft}}=P_{\text{rescue}}-P_{\text{hard}}.$$ (6)

**Figure 3:**
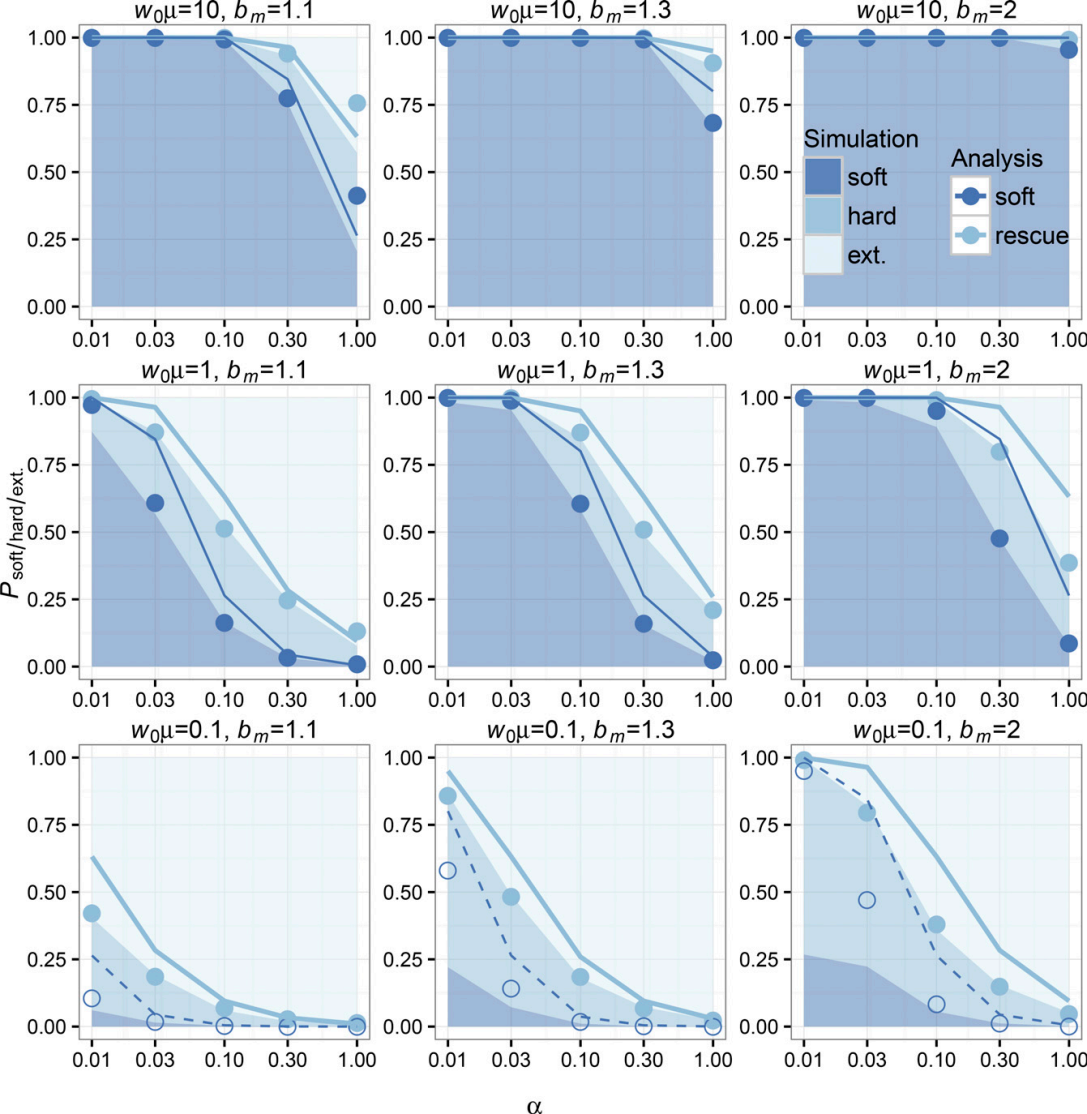
Simulations and analytic predictions for evolutionary rescue in low-density scenarios. The probabilities of observing rescue via soft selective sweeps, rescue via hard selective sweeps, or extinction as a function of the decline rate *α* (logarithmic scale) measured over 1000 simulations (see *Methods*) for each combination of model parameters are indicated as stacked bar plots. The color key indicates shading for simulation outcomes. Population-scale mutation rate increases between plots from bottom to top, and unscaled per-capita mutant birth rate increases between plots from left to right. The analytic predictions for each parameter combination show $P_{\text{soft}}$ (bottom blue points) and $P_{\text{rescue}}=P_{\text{hard}}+P_{\text{soft}}$ (top, light blue points). Our Poisson approximation is indicated using lines of the same colors. For analytic predictions and our Poisson approximation of $P_{\text{soft}},$ points where $w_{0}\mu \geq 1$ are shaded and lines are solid. Our analysis has high concordance with the observed probability of rescue for each parameter combination. Our analysis also has high concordance with the observed probability of rescue via soft sweeps for most parameter combinations, except in instances where our independence assumption breaks down $\left(w_{0}\mu <1\right)$ for low decline rates (bottom row, leftmost *α* values).

**Figure 4:**
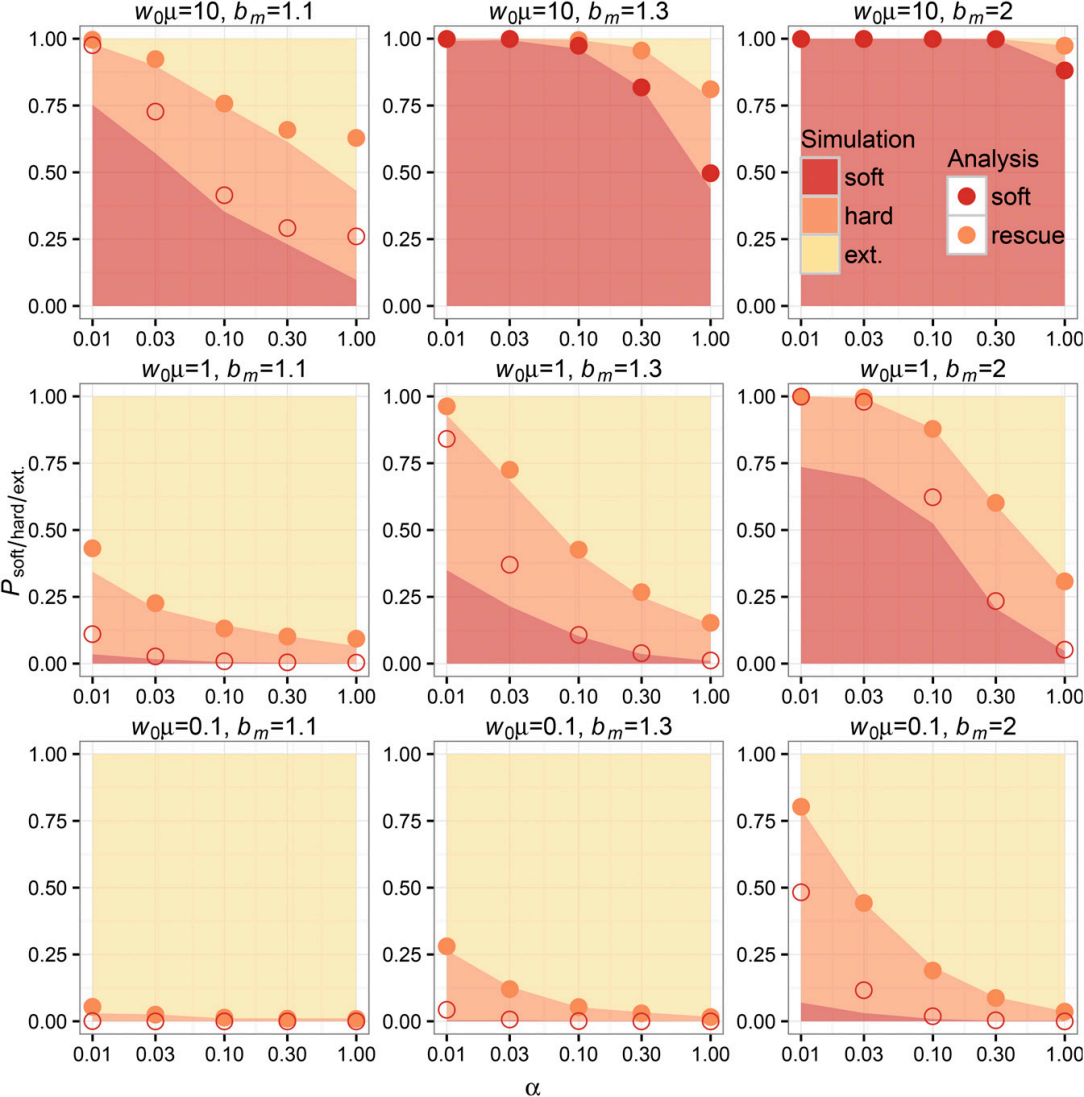
Simulations and analytic predictions for evolutionary rescue in high-density scenarios. The probabilities of observing rescue via soft selective sweeps, rescue via hard selective sweeps, or extinction as a function of the decline rate *α* (logarithmic scale) measured over 1000 simulations (see *Methods*) for each combination of model parameters are indicated as stacked bar plots. The color key indicates shading for simulation outcomes. Population-scale mutation rate increases between plots from bottom to top, and unscaled per-capita mutant birth rate increases between plots from left to right. The analytic predictions for each parameter combination show $P_{\text{soft}}$ (bottom red points) and $P_{\text{rescue}}=P_{\text{hard}}+P_{\text{soft}}$ (top orange points). For analytic predictions of $P_{\text{soft}},$ points where adaptation w*μ≥1 are shaded. Our analysis has high concordance with the observed probability of rescue for each parameter combination. As in the low-density scenario, our analysis has good concordance with the observed probability of rescue via soft sweeps for most parameter combinations, except in instances where our independence assumption breaks down (w*μ<1) and for low decline rates (bottom and middle rows, leftmost *α* values). Evolutionary rescue is less likely for more parameter combinations in the high-density scenario because the wild-type population must decline before establishment of mutants is likely (see Figure 2).

To confirm our analysis, we performed forward-time, birth-death simulations in populations initially comprised of 10,000 wild-type individuals over multiple values of *K*, *α*, $b_{m},$ and *μ* (see *Methods* section for details). If we examine $P_{\text{rescue}}$ for low-density $(w_{0}=10,000,$ K=110,000) and high-density $(w_{0}=10,000,$ K=10,000) rescue scenarios, we can see that the overall probabilities of rescue and rescue via soft sweeps decline with increasing *α* (see simulation and analytic values from Equations 4 and 6 plotted in Figure 3 and Figure 4). The qualitative dependence on decline rate in our model is the same as seen in previous models where mutation was weak (Orr and Unckless 2008, 2014). As for the other relevant parameters in our model, the probabilities of evolutionary rescue and rescue via soft sweeps increase universally with increasing *μ* and $b_{m}.$ Rescue is generally higher in low-density scenarios (when the carrying capacity is higher than the wild-type population size) than in high-density scenarios (when the carrying capacity is close to the wild-type population size), similar to simulation results for scenario 2 under “Alternative Forms of Population Regulation” in Orr and Unckless (2008). We also find that sweeps are generally softer in low-density scenarios than in high-density scenarios.

Simulations and analysis agree well for both low- and high-density scenarios when comparing corresponding values of $P_{\text{rescue}}.$ Our analysis also agrees with the observed probability of rescue via soft sweeps in scenarios where mutation is high and mutants have an appreciable chance of establishment (see Figure 3 and Figure 4). In instances where mutation is rare (such as when $w_{0}\mu <1$ for low-density rescue or when the population must decline dramatically before mutants can establish, such that w*μ<1 for high-density rescue) and where the wild-type population declines slowly, our analytic assumption regarding the independence of mutant lineages during establishment breaks down; resulting in deviations from the values observed in simulations. In particular, our analysis overestimates the probability of soft sweeps because it excludes the contribution of the mutant subpopulation in the density-dependent term in Equation 1. See *Evolutionary rescue via hard selective sweeps* for further explanation.

### Simple Poisson approximation for low-density rescue

From Equation 3 follows that, when $d_{m}=1$ and when the mutant birth rate is not much higher than 1, the establishment probability asymptotes to $b_{m}-1.$ As can be seen in Figure 2A, when the population density is low, this asymptote is reached quickly. It is therefore possible to derive a simple approximation under the assumption that $p_{\mathrm{est}.}=b_{m}-1,$ independent of *t*. When the initial wild-type population size is $w_{0}$ and the wild-type population declines at rate *α*, then the total number of individuals in the population until extinction is expected to be $w_{0}/\alpha .$ If we multiply this with the mutation rate and the establishment probability, we find the expected number of successful mutants to be $k=\left(w_{0}\mu /\alpha \right)\left(b_{m}-1\right).$ Under the assumption that mutations are independent, the realized number of successful mutants will follow a Poisson distribution with rate parameter

$$\Lambda =\frac{w_{0}\mu \left(b_{m}-1\right)}{\alpha }.$$ (7)

Because Λ is equal to the expected value for Poisson random variables, it is easy to see from this relationship that the expected number of beneficial mutants that survive extinction increases with increasing $w_{0}\mu$ and $b_{m},$ and decreases with increasing *α*. This approximation is illustrated in Figure 3 and generally gives slight overestimates to the probabilities of rescue $\left[P_{\text{rescue}}=P\left(k\geq 2\right)\right]$ and rescue via soft sweeps $\left[P_{\text{soft}}=P\left(k\geq 1\right)\right]$ seen in simulations, which is expected because the approximation uses the highest establishment probability for all *t*.

### Soft sweeps are more likely when rescue is likely

**Figure 5:**
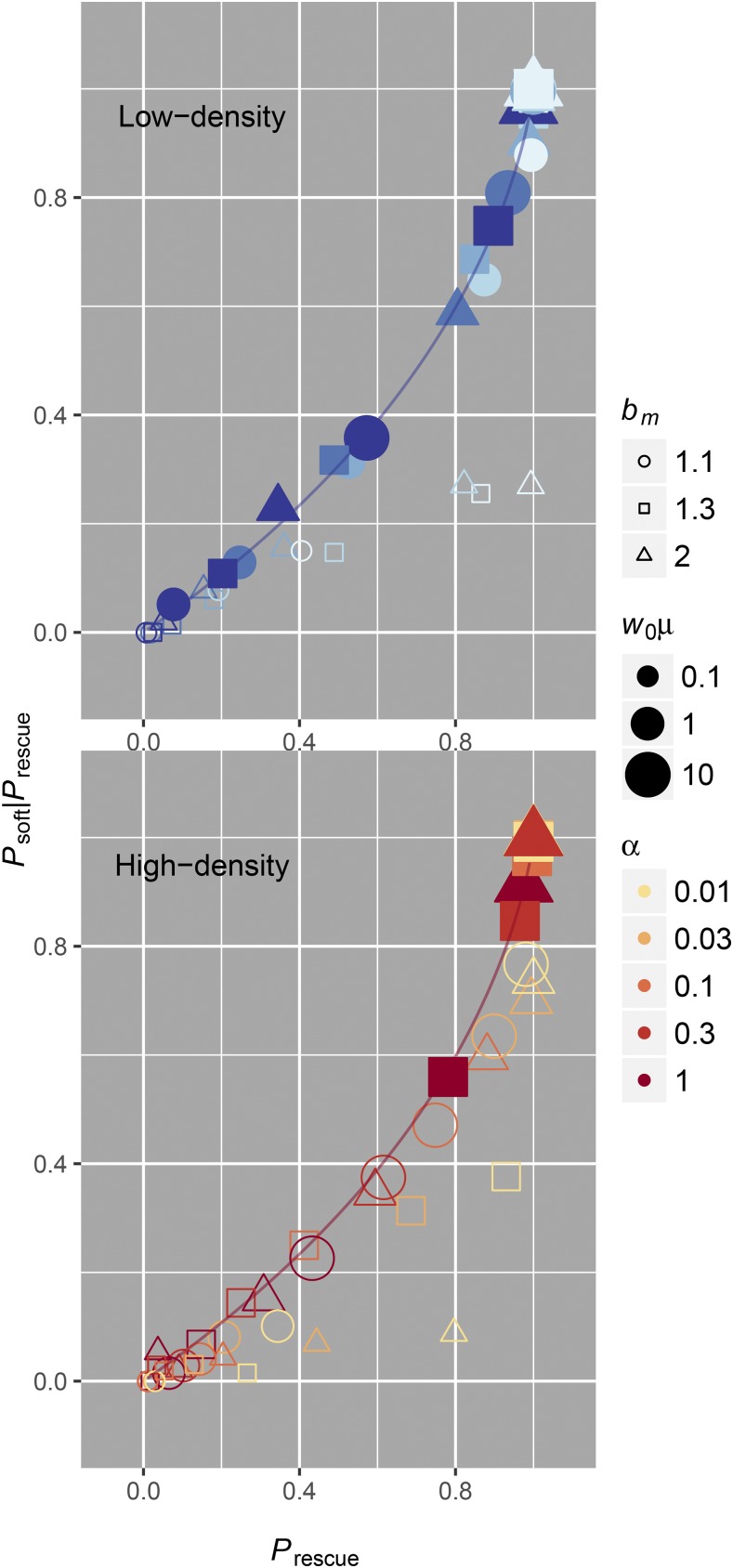
When rescue is likely it is driven by soft sweeps. Both in low-density (top, blue) and high-density (bottom, red) scenarios, simulations (points) indicate that soft sweeps are more prevalent when evolutionary rescue is likely. The correlation lines assume independence between lineages and are plotted according to the relationship in Equation 8. We have only shaded simulations where our independence assumption is valid $[w_{0}\mu \geq 1$ in low-density rescue and w*μ≥1 in high-density rescue, where $w*=K\left(1-d_{m}/b_{m}\right)].$ When mutation is rare, we expect our modeling assumptions to fail or soft sweeps to be unlikely. Departures from independence are more pervasive in high-density situations, especially when decline rates are slow.

Both $P_{\text{rescue}}$ and $P_{\text{soft}}$ vary similarly with the underlying parameters of our model because they both strongly depend on the area under the intensity function R(t). If we ask whether sweeps are more likely to be soft conditional on rescue occurring, we can thus see an obvious correlation between the two phenomena. This correlation is shown in Figure 5, where $P_{\text{soft}}|P_{\text{rescue}}$ is plotted against $P_{\text{rescue}}.$ Mathematically, we can derive the relationship using Equations 4–6. First, solving $P_{\text{hard}}$ in terms of $P_{\text{rescue}}$ yields $P_{\text{hard}}=-\left(1-P_{\text{rescue}}\right)\log\left(1-P_{\text{rescue}}\right).$ Solving for $P_{\text{soft}}|P_{\text{rescue}}$ in terms of $P_{\text{rescue}}$ then gives

$$\begin{array}{c} P_{\text{soft}}|P_{\text{rescue}}=P_{\text{soft}}/P_{\text{rescue}}\\ =\left(P_{\text{rescue}}-P_{\text{hard}}\right)/P_{\text{rescue}}\\ =\left[P_{\text{rescue}}+\left(1-P_{\text{rescue}}\right)\log\left(1-P_{\text{rescue}}\right)\right]/P_{\text{rescue}}\\ =1+\left(\frac{1-P_{\mathrm{rescue}}}{P_{\mathrm{rescue}}}\right)\log\left(1-P_{\text{rescue}}\right), \end{array}$$ (8)

which is monotonically increasing for $P_{\text{rescue}}$ in (0,1).

While it may be intuitive that the probabilities of rescue and soft sweeps are correlated (both the probability of rescue and the probability of soft sweeps depend on the same rate parameter and increase with the number of mutations that occur), we highlight it because of its relevance to postrescue genetic diversity. The hallmark of a soft selective sweep is that multiple lineages are preserved after selection (Hermisson and Pennings 2005; Pennings and Hermisson 2006a). This means that selection need not remove all genetic diversity in a population following evolutionary rescue, especially when rescue is expected to be common. We discuss why this might be important in the *Discussion*.

### Evolutionary rescue via hard selective sweeps

Although the primary focus of this article is to investigate soft selective sweeps in evolutionary rescue, we feel that evolutionary rescue via hard sweeps deserves special attention. When mutations are rare, the population will typically either go extinct or be rescued by a single mutant lineage via a hard selective sweep. Neither of these outcomes should affect the validity of our assumption of independence between lineages in Equation 1 because they involve either zero or one mutant lineages, respectively. However, in scenarios where the wild-type population declines very slowly and where the time between mutant establishments is long, it is possible that one mutant lineage establishes and reaches a size large enough to prevent a second mutant lineage from establishing, leading to a hard sweep. This scenario is not accounted for in our analysis because we exclude the mutant contribution to population density in the density-dependent term in Equation 1. In these situations, the window of opportunity for a second mutant lineage to establish is limited by the time it takes for the first established mutant lineage to bring the total population size high enough to substantially decrease the establishment probability of a second mutant lineage; assuming the wild-type population declines slowly enough to allow multiple mutations to appear before it goes extinct. This is similar to the scenario in Pennings and Hermisson (2006a) where the establishment of a second mutant was limited by the time it takes the first mutant to sweep in a population. What is most noteworthy about these situations is that whether evolutionary rescue occurs via soft sweeps depends not only on the population-scale mutation rate at the onset of the environmental shift, but also on the population density at that point in time. Departures from our independence assumption are more frequent in the high-density scenarios than in the low-density scenarios that we explored. This is because in high-density scenarios, mutant lineages are unlikely to establish until the wild-type population declines to $w*∼K\left(1-d_{m}/b_{m}\right),$ at which point the population-scale mutation rate can be small (w*μ<1) even when the initial population-scale mutation rate was large $\left(w_{0}\mu \geq 1\right).$ Understanding when we expect nonindependence and when we expect evolutionary rescue to appear via soft sweeps is therefore dependent on approximating the population-scale mutation rate when mutant lineages have an appreciable probability of establishing. We have distinguished scenarios where mutant-establishment probabilities are approximately independent $(w_{0}\mu \geq 1$ in low-density rescue and w*μ≥1 in high-density rescue) from scenarios where our assumptions regarding independence between lineages are expected to break down $(w_{0}\mu <1$ in low-density rescue and w*μ<1 in high-density rescue) in Figure 3 and Figure 4. While this heuristic distinction is conservative and does not account for differences in decline rate, we find that it provides a clean partition of where our analysis performs well (shaded points in in Figure 3 and Figure 4) and where it does not (open points in in Figure 3 and Figure 4).

### Waiting-time distributions for the establishment of mutants

It may be of interest to reformulate the results from our previous analysis in terms of the waiting times associated with the establishment of the individual adaptive lineages during the extinction process. As in the previous analysis, we will assume independence between mutant lineages. Under this assumption, we define $\tau_{1}$ to be the waiting time for the first mutant lineage to establish. $\tau_{1}$ has probability density equal to

$$p\left(\tau_{1}\right)=R\left(\tau_{1}\right)\exp\left[-\int_{0}^{\tau_{1}}R\left(t\right)dt\right],$$ (9)

conditional on evolutionary rescue occurring. Note that this is identical to equation A8 in Uecker and Hermisson (2011) with $P_{\text{rescue}}=1.$ Conditional on the establishment of a first adaptive mutant at $\tau_{1},$ the probability density for the establishment of a second adaptive mutant at time $\tau_{2}$ takes the same form integrated over all possible $\tau_{1}.$ The probability density for $\tau_{2}$ is

$$p\left(\tau_{2}\right)=\int_{0}^{\tau_{2}}p\left(\tau_{1}\right)R\left(\tau_{2}\right)\exp\left[-\int_{\tau_{1}}^{\tau_{2}}R\left(t\right)dt\right]d\tau_{1}.$$ (10)

**Figure 6:**
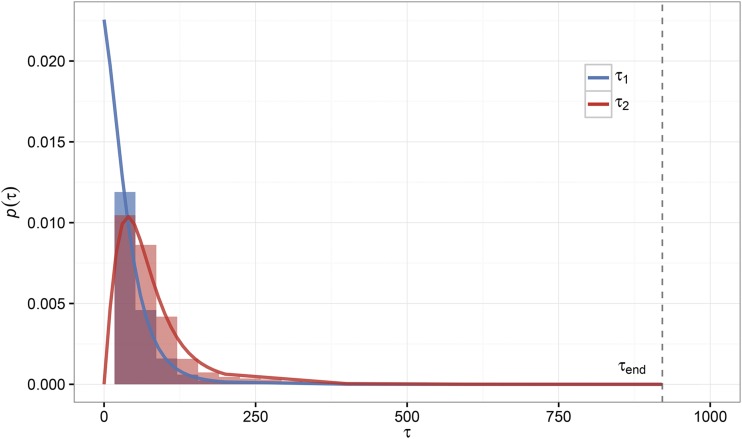
Waiting-time distributions for the establishment of the first and second mutants during rescue. Shown are the probability densities for the first and second mutants to establish during evolutionary rescue according to Equation 9 (blue line) and Equation 10 (red line). Empirical distributions from 10,000 simulations are shown in the same corresponding colors but as density histograms. The particular scenario is a low-density rescue scenario with $w_{0}=10,000,$ K=110,000, α=0.01,$b_{m}=1.1,$ and $w_{0}\mu =1.$

Distributions for both $\tau_{1}$ and $\tau_{2}$ are plotted for one set of parameters in Figure 6. Equations 9 and 10 are in good agreement with forward-time, birth-death simulations for this parameter combination; although it is important to consider the previous discussion regarding the independence of mutant lineages during establishment and the regime where independence breaks down, in which case we expect departures for the distribution for $\tau_{2}$ but the distribution for $\tau_{1}$ should remain unchanged. In theory, we could use the probability density of $\tau_{1}$ to approximate a trajectory $m\left(\tau_{1}\right)$ for the first established mutant, which could then be used to calculate an establishment probability for a second mutant that includes the previous mutant’s contribution to population density. However, we find this to be unnecessarily convoluted in practice.

## Discussion

We show that adaptation often proceeds via soft selective sweeps when evolutionary rescue is likely. Our results regarding the probability of evolutionary rescue agree with results previously published using similar models of adaptation under *de novo* mutation variation (Orr and Unckless 2008, 2014; Uecker and Hermisson 2011; Martin *et al.* 2013; Uecker *et al.* 2014).

### The generality of soft sweeps in evolutionary rescue

We study soft selective sweeps via *de novo* mutation as a mode of evolutionary rescue because of their relevance to case studies of adaptation, and particularly adaptation to strong environmental pressures (Karasov *et al.* 2010; Messer and Petrov 2013; Pennings *et al.* 2014; Feder *et al.* 2016). While adaptation from standing genetic variation is also expected to generate soft sweeps (Hermisson and Pennings 2005), it may be the case that adaptive mutants are absent at the onset of an environmental shift because they are strongly deleterious in the prior environment, as can be the case in resistance evolution (Andersson 2003; Shi *et al.* 2004; Cong *et al.* 2007). In reality, both modes of adaptation will play a role in the process of evolutionary rescue, and evolutionary rescue will depend strongly on the underlying ecological and population-genetic factors of the adapting population, such as population density, population substructure, epistasis, and genetic recombination. For example, whether fast decline of maladapted individuals in the population inhibits or facilitates evolutionary rescue depends strongly on whether adaptive mutations already exist in the population and whether there is strong population substructure (Wargo *et al.* 2007; Gatenby *et al.* 2009; Read *et al.* 2011; Uecker *et al.* 2014). Even in unstructured populations, fast decline of the wild-type population can increase the probability of rescue from standing genetic variation while decreasing the probability of rescue from *de novo* mutation, leading to a nonmonotonic dependence on decline rate when both adaptive processes are present (see appendix B in Uecker *et al.* 2014). And whether complex adaptations that require multiple mutations facilitate evolutionary rescue when a population faces an environmental challenge is strongly dependent on epistatic interactions between mutations and the presence (or absence) of genetic recombination (Lindsey *et al.* 2013; Uecker and Hermisson 2016). Nevertheless, modeling evolutionary rescue as a Poisson process in each of these complex scenarios has led to a general form for to the probability of rescue as $P_{\mathrm{rescue}}=1-\exp\left(-\Lambda \right),$ where Λ is the number of expected mutants generated via *de novo* mutation, or existing in standing variation, that are expected to survive extinction. While this number is a complex function of the aforementioned ecological and population-genetic factors, the probability is nonetheless always higher when the number of surviving mutants is higher. Our choice of a *de novo* mutation model in this article, while simple, is meant to illustrate this without unnecessary complications. Previous empirical observation (Bell and Gonzalez 2009) and intuition therefore suggest that when rescue is likely, more adaptive mutants are expected to be involved. It is for this reason that we expect soft sweeps to be a general feature of evolutionary rescue in situations where it is most likely to occur. Conversely, our message could be flipped to conclude that in cases where extinction is likely, evolutionary rescue (should it occur) will occur via hard selective sweeps.

Our model captures important aspects of population dynamics and natural selection, although there are some limitations that we feel should be addressed. First, departures from our analytic assumptions occur in scenarios where mutations are rare and when population decline is very slow, namely establishment of one mutant lineage will affect the establishment of subsequent mutant lineages because of density-dependent mutant growth rates. Though we have chosen to not explicitly model this particular regime because it is not related to our primary focus on high recurrent mutation and soft sweeps, it is noteworthy to consider how populations can produce frequent rescue via hard sweeps when population decline is slow (illustrated in Figure 3, Figure 4, and Figure 5).

**Figure 7:**
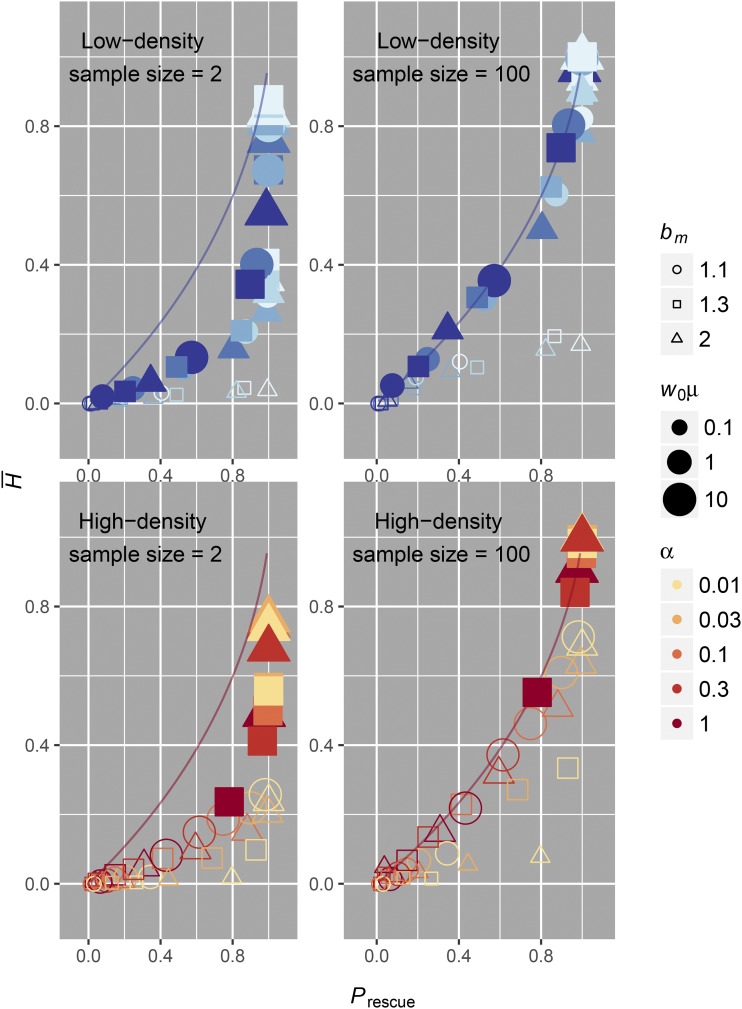
Mean heterozygosity from simulations of evolutionary rescue. Heterozygosity of the rescued population was calculated for each simulation and is equivalent to the probability that all sampled individuals do not come from a single mutant lineage. Plotted is the mean heterozygosity $\left(\bar{H}\right)$ averaged over all 1000 simulations for each parameter set against the corresponding probability of rescue for each parameter set. Only parameter sets where our independence assumptions are valid $(w_{0}\mu \geq 1$ for low-density scenarios and w*μ≥1 for high-density scenarios) are shaded. Although the sample depth is as low as possible for a meaningful measure of genetic diversity in the “sample size = 2” case, we still see a general correlation between genetic diversity and probability of rescue. The sensitivity with which one could distinguish rescue probability using genetic diversity is much smaller compared to the previous measure of $P_{\text{soft}},$ where we knew the precise number of lineages following evolutionary rescue. However, the sensitivity is virtually identical for larger samples, as seen in the “sample size = 100” case. For larger sample sizes, the values of mean heterozygosity inferred from our simulations more closely match our analytic predictions (colored lines). This indicates that deeper population samples will have better sensitivity toward identifying whether rescue was likely or unlikely in scenarios where it is otherwise difficult to ascertain the probability of rescue.

Second, another drawback of our analysis is that our measure of $P_{\text{soft}}$ is connected to the establishment of adaptive lineages during the process of evolutionary rescue (inferred from knowledge of the population composition after rescue or extinction) and not specifically connected to a sample genealogy, as in Pennings and Hermisson (2006a) and Wilson *et al.* (2014). This means that our measure of $P_{\text{soft}}$ will not necessarily capture how the probability of observing a soft selective sweep depends on lineage frequencies. It is possible that lower frequency lineages could be missed in shallow samples. We can see how the observed relationship between average heterozygosity $\left(\bar{H}\right)$ in our simulations (measured as the probability that all mutants sampled immediately following rescue are not identical by descent) and rescue probability has the same basic correlation as our measure of $P_{\text{soft}}|P_{\text{rescue}}$ in Figure 7. There is lower sensitivity to detect genetic diversity in such shallow samples (sample size = 2 in Figure 7), but in larger samples, the expected correlation is virtually identical to the analytic expectation (sample size = 100 in Figure 7). We therefore highlight that empirical observations of soft selective sweeps in rescued populations are still crucially dependent on sample depth.

Finally, we note that in this article we have only considered the locus where the beneficial mutation that leads to rescue can occur, and we have not considered any neutral loci in the genome, whether linked to the selected locus or not. Whether a selective sweep in a rescued population can be detected is a question that deserves separate treatment, but it is likely that selective sweeps will be very hard to detect if the population as a whole goes through a severe bottleneck. This could mean that in rescued populations, hard sweeps may be harder to detect than soft sweeps because hard sweeps tend to occur when the population bottleneck is more severe.

### The importance of genetic diversity in rescued populations

In Orr and Unckless (2014), the authors discuss how the average minimum population size that is reached during evolutionary rescue is smaller for adaptation via *de novo* mutation than for adaptation from standing genetic variation because of the dependence on the waiting time for the first established mutant $(\tau_{1}$ in this article). They posit that this may lead to lower genetic diversity in populations rescued via *de novo* mutation than those rescued from standing genetic variation, presumably because a larger average minimum population size would reduce the strength of genetic drift, increase the average population-scale mutation rate, and provide more opportunities for recombination to generate diversity within the rescued population. However, this relationship between genetic diversity and the mode of adaptation (from standing *vs.* *de novo* variation) is not as direct when adaptation is driven by soft sweeps. Adaptation via soft sweeps may not drastically remove genetic diversity in an adapting population (Pennings and Hermisson 2006b), as might be expected in a hard sweep where only one lineage carries the adaptive mutation. In rescue via soft sweeps, adaptive mutations occur on different genetic backgrounds either before the environmental shift (in adaptation from standing genetic variation) or during the population decline after the environmental shift (in adaptation via *de novo* mutation). The degree to which each mode of adaptation reduces genetic variation will depend on the number of genetic backgrounds on which the adaptive mutation occurs. The number of different lineages could even be larger for adaptation via *de novo* mutation if the adaptive mutation is strongly deleterious before the environmental shift (explicitly, if fewer mutants would be drifting in deleterious mutation-selection balance than would establish during the population decline). The number of adaptive lineages could be higher for adaptation from standing genetic variation if the adaptive mutation is already segregating on many different genetic backgrounds before the environmental shift. In either case, soft sweeps will play a significant role in preserving genetic diversity in the adapting population.

There are multiple reasons why higher genetic diversity following evolutionary rescue might be an important consideration. First, preserving genetic diversity that was present prior to the environmental shift will be important to the future fitness of the population following evolutionary rescue, especially in populations that cannot generate diversity quickly. If evolutionary rescue occurs via soft selective sweeps, then some of this ancestral diversity will be maintained for future generations. Second, postrescue genetic diversity can be a useful proxy for measuring how likely such a population was to adapt to an environmental pressure. For example, genetic diversity in the viral population after the emergence of drug resistance could be used to determine the efficacy of a drug used to treat a virus within a patient in higher resolution than viral load alone, because genetic diversity is expected to correlate with the likelihood that treatment failure occurred *a priori*. In other words, when treatment failure is common, $P_{\text{rescue}}$ is highest; and when $P_{\text{rescue}}$ is highest, we expect treatment failure to be driven by soft sweeps (Feder *et al.* 2016). This leads to higher genetic diversity in samples where failure was common and driven by soft sweeps than in samples where failure was rare and driven by hard sweeps. Indeed, others have found a correlation between treatment efficacy in HIV and whether treatment failure occurred via hard or soft sweeps that is in agreement with the theoretical results of this article (Feder *et al.* 2016). Knowledge of this expected correlation may be broadly applicable to analysis of other types of drug-resistant infections, such as malaria. This correlation between genetic diversity and the likelihood of evolutionary rescue is expected to decay over time in an asexual population, as genetic drift will eventually remove all but one lineage; so the timing of the population sample will be critical to the assessment of the likelihood of evolutionary rescue.
